# Supplementary material for: Mesopelagic Species and Their Potential Contribution to Food and Feed Security—A Case Study from Norway
Source: Foods. 2020 Mar 16;9(3):344. doi: 10.3390/foods9030344 (PMC7142554; doi:10.3390/foods9030344)
Supplement: Supplementary file 1 [file foods-09-00344-s001.zip › Supplementary_Foods.docx]

**Supplementary Material**

**Table S1:** **Potential amount of protein, fat and selected micronutrients in 6 mesopelagic species per km^3^ of Oster- and Bjørnafjorden in Norway**

|  | **Protein** (kg/km^3^) | **Fat**  (kg/km^3^) | **Vitamin A1** (g/km^3^) | **Iodine** (g/km^3^) | **Ca** (kg/km^3^) | **Na** (kg/km^3^) | **K**  (kg/km^3^) | **Mg** (kg/km^3^) | **P**  (kg/km^3^) | **Fe** (g/km^3^) | **Zn** (g/km^3^) | **Se**  (g/km^3^) |
| --- | --- | --- | --- | --- | --- | --- | --- | --- | --- | --- | --- | --- |
| *Benthosema glaciale* | 1560 | 1530 | 183 | 5 | 56 | 43 | 29 | 8 | 43 | 121 | 95 | 7 |
| *Maurolicus muelleri* | 500 | 720 | 41 | 1 | 22 | 15 | 9 | 2 | 16 | 64 | 46 | 2 |
| *M. norvegica* | 942 | 336 | 4 | 7 | 40 | 30 | 22 | 10 | 22 | 130 | 61 | 6 |
| *Pasiphaea sp.* | 128 | 49 | 0 | 0.4 | 6 | 3 | 3 | 1 | 3 | 4 | 8 | 0.4 |
| *Eusergestes arcticus* | 577 | 351 | 1 | 4 | 20 | 14 | 13 | 3 | 14 | 12 | 67 | 2 |
| *Periphylla periphylla* | 2965 | 1405 | 1 | 7 | 134 | 3121 | 259 | 328 | 38 | 137 | 328 | 12 |
| Total | 6787 | 4501 | 244 | 25 | 282 | 3230 | 337 | 352 | 140 | 477 | 611 | 30 |

**Table S2: Potential amount of selected macrominerals (Ca, Na, K, Mg, P) and micronutrients in the protein (Fe, Zn, Se) and in oil fraction (vitamin A_1_) from 6 mesopelagic species of Oster- and Bjørnafjorden in Norway on wet weight basis.**

|  | **Protein fraction** | | | | | | | | | | | **Oil fraction** |
| --- | --- | --- | --- | --- | --- | --- | --- | --- | --- | --- | --- | --- |
|  | | |  | **Ca**  (g/100g) | **Na**  (g/100g) | **K** (mg/100g) | **Mg** (mg/100g) | **P** (mg/100g) | **Fe** (mg/100g) | **Zn**  (mg/100g ) | **Se**  (µg/100g) | **Vitamin A1** (µg/100g) |
| *Benthosema glaciale* | | |  | 3600 | 2800 | 1900 | 480 | 2700 | 8 | 6 | 440 | 12000 |
| *Maurolicus muelleri* | | |  | 4400 | 3100 | 1800 | 500 | 3200 | 13 | 9 | 360 | 5700 |
| *M. norvegica* | | |  | 4200 | 3200 | 2300 | 1100 | 2400 | 14 | 6 | 650 | 1100 |
| *Pasiphaea sp.* | | |  | 4500 | 2400 | 2000 | 590 | 2400 | 3 | 7 | 310 | 200 |
| *Eusergestes arcticus* | | |  | 3400 | 2300 | 2300 | 480 | 2400 | 2 | 12 | 330 | 370 |
| *Periphylla periphylla* | | |  | 4500 | 110000 | 8700 | 11000 | 1300 | 5 | 11 | 400 | 67 |
| Average catch composition | | w.o jellies | | 3900 | 2800 | 2000 | 620 | 2700 | 9 | 7 | 460 | 7700 |
|  |  | with jellies | | 4300 | 61000 | 5900 | 6600 | 1900 | 6 | 10 | 430 | 4300 |

It was assumed that all minerals follow the protein fraction and vitamin A the oil fraction. w.o = without

**Table S3: Relative amounts of wax esters in *B. glaciale* and *E. arcticus***

|  | *Benthosema glaciale*  %  (min – max)  n = 7 | *Eusergestes arcticus*  %  (min – max)  n = 3 |
| --- | --- | --- |
| **14:0 ALK** | 4.3 ± 0.7  (3,6 – 6,1) | 4.8 ± 1.8  (2,9 – 6,9) |
| **16:0 ALK** | 26.0 ± 1.7  (23,9 – 29,2) | 20.2 ± 6.5  (14,1 – 29,1) |
| **18:0 ALK** | 2.6 ± 0.5  (1,8 -3,4) | 1.2 ± 0.5  (0,8 – 1,8) |
| **SUM SFA** | 34.3 ± 2.0  (32,6 – 38,9) | 27.6 ± 8.4  (19,2 – 38,0) |
| **16:1 ALK** | 3.1 ± 0.1  (2,9 – 3,3) | 5.1 ± 1.1  (3,7 – 6,1) |
| **18:1 ALK** | 11.8 ± 2.6  (7,5 -15,4) | 4.1 ± 1.3  (3,1 – 6,0) |
| **20:1 ALK** | 15.0 ± 1.7  (12,4 – 17,6) | 17.6 ± 2.7  (13,6 – 19,4) |
| **22:1 ALK** | 29.7 ± 3.3  (23,4 – 34,0) | 41.3 ± 8.1  (30,9 – 50,0) |
| **SUM MUFA** | 63.7 ± 2.2  (58,5 – 65,4) | 70.6 ± 8.5  (60,0 – 79,2) |
| **18:2n-6 ALK** | 1.0 ± 0.1  (0,9 – 1,4) | 0.9 ± 0.1  (0,8 – 1,0) |
| **18:3n-3 ALK** | 0.8 ± 0.1  (0,6- 1,0) | 0.6 ± 0.2  (0,5 – 0,8) |
| **SUM PUFA** | 2.0 ± 0.1  1,8 – 2,6) | 1.7 ± 0.2  (1,6 – 1,9) |


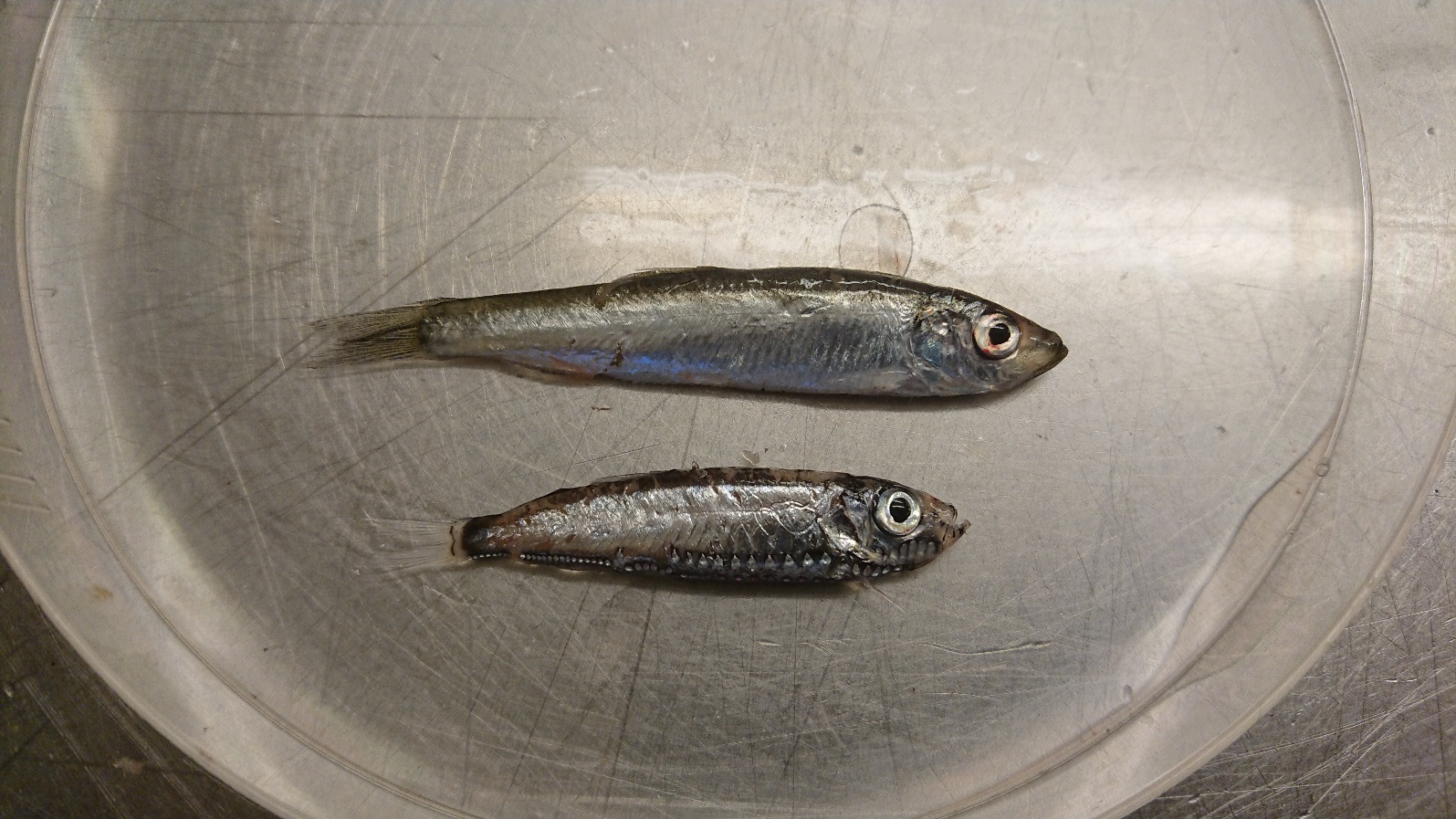


**Figure S1:** The mesopelagic fish species silvery lightfish (*Maurolicus muelleri*, bottom) is similar to sprat (*Sprattus sprattus*, top), a commonly consumed fish, both in appearance and nutrient composition. Both fish were caught in the same catch. Photo by Martin Wiech.


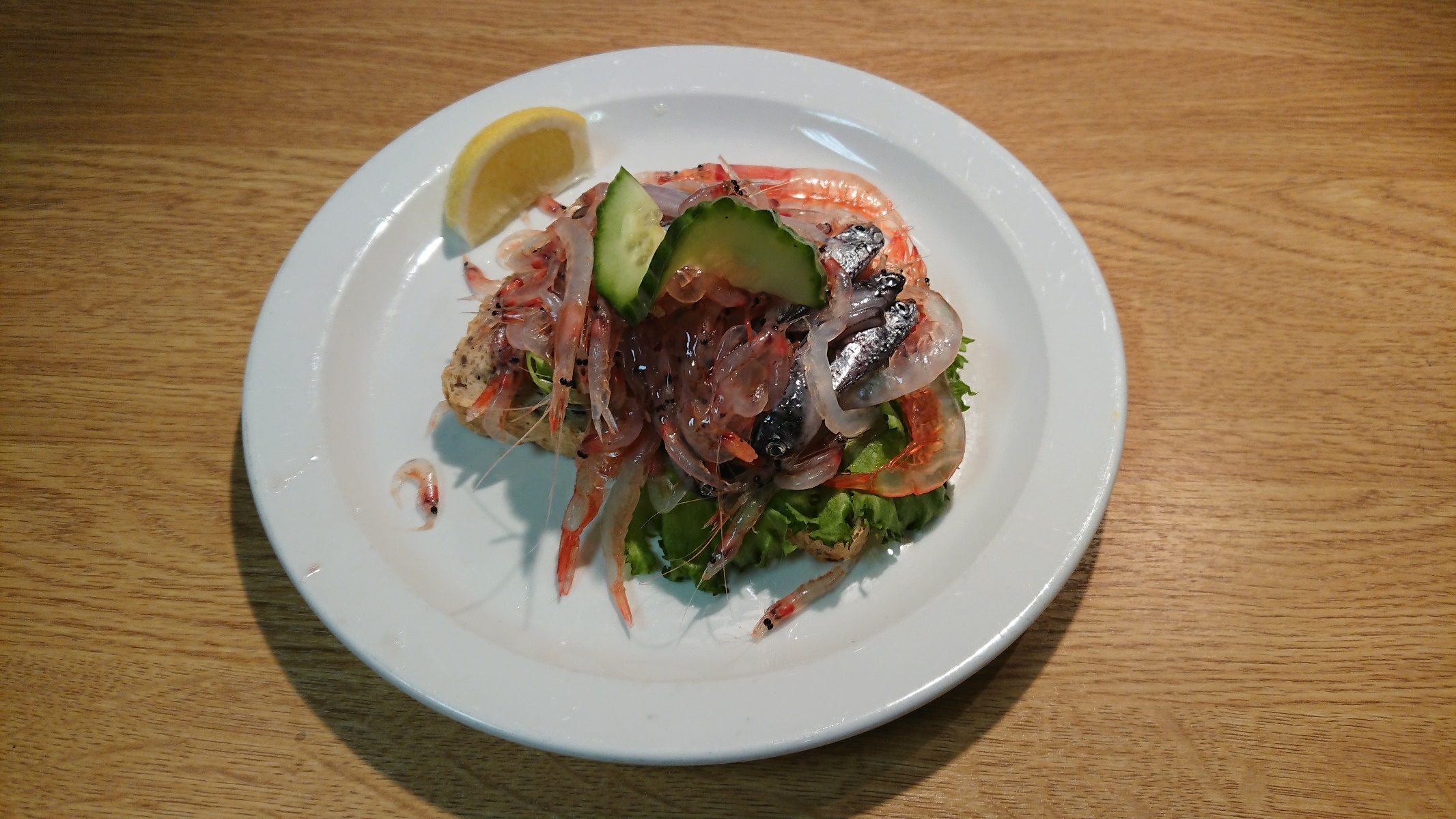


**Figure S2:** Dietary potential of mesopelagic biomass. Several mesopelagic species as sandwich spread. Photo by Martin Wiech
